# Effects of Statins on Incident Dementia in Patients with Type 2 DM: A Population-Based Retrospective Cohort Study in Taiwan

**DOI:** 10.1371/journal.pone.0088434

**Published:** 2014-02-10

**Authors:** Jui-Ming Chen, Cheng-Wei Chang, Tzu-Hao Chang, Chi-Chang Hsu, Jorng-Tzong Horng, Wayne H-H Sheu

**Affiliations:** 1 Department of Endocrinology and Metabolism, Tungs' Taichung MetroHarbor Hospital, Taichung, Taiwan; 2 Department of Information Management, Hsing Wu University, New Taipei City, Taiwan; 3 Department of Biomedical Informatics, Asia University, Taichung, Taiwan; 4 Department of Gerontechnology and Service Management, Nan Kai University of Technology, Nantou, Taiwan; 5 Graduate Institute of Biomedical Informatics, Taipei Medical University, Taipei, Taiwan; 6 Department of Computer Science and Information Engineering, National Central University, Chungli, Taiwan; 7 Division of Endocrinology and Metabolism, Department of Internal Medicine, Taichung Veterans General Hospital, Taichung, Taiwan; 8 School of Medicine, National Defense Medical Center, Taipei, Taiwan; 9 School of Medicine, National Yang-Ming University, Taipei, Taiwan; 10 Institute of Medical Technology, National Chung-Hsing University, Taichung, Taiwan; “Mario Negri” Institute for Pharmacological Research, Italy

## Abstract

**Background:**

Patients with Type 2 diabetes (T2DM) are prone to develop dementia. Results from a recent study indicated that statin users had lower chance of developing incident dementia. However there is little information on the potential benefits of statin use on dementia in patients with T2DM cohort.

**Method:**

A population-based retrospective study using a nationwide cohort of National Health Insurance Research Database in Taiwan was performed. T2DM cohort with regular use of statins was followed up to 8 years. Multivariate cox-proportional hazards regression model was used to estimate the association between statin use and incidence of dementia including Alzheimer's disease and non-Alzheimer dementia after adjusting for several potential confounders.

**Results:**

Among 28,321 patients diagnosed with T2DM age above 50 and without history of dementia before 2000/1/1, 15,770 patients who had never used statin and 2,400 patients who regularly used statin drugs were enrolled. After adjusting for age group, gender, CCI (Charlson-Deyo comorbidity index) group, stroke types and anti-diabetic drugs, regular statin use was associated with a decreased risk of developing incident Alzheimer's disease dementia (adjusted HR: 0.48, 95% CI 0.30 – 0.76, p<0.001), but not in non-Alzheimer dementia (adjusted HR: 1.07, 95% CI 0.54–2.12 p = 0.844) in patients with T2DM. Further analysis showed significant protective effects of the use of atorvastatin and simvastatin.

**Conclusion:**

Regular use of statins might decrease the risk of developing Alzheimer's disease in patients with T2DM while no benefit was observed in non-Alzheimer dementia. Among statins, both atorvastatin and simvastatin showed significant benefits.

## Introduction

It is now well recognized that people with diabetes have a greater decline in cognitive function and a higher risk of developing dementia [1], although some studies declared that the relation between diabetes and major types of dementia remains controversial [2]. Notably, a large body of researches reported that diabetes may increase the risk of Alzheimer's disease as well as vascular dementia in both sexes and in all ages [3]–[6].

Results from an early database analysis from UK indicated that Individuals of 50 years and older who were prescribed statins had a substantially lowered risk of developing dementia, although their study did not distinguish Alzheimer's disease and other forms of dementia [7]. Since then, a number of epidemiological studies had examined the effects of statin use in the prevention and treatment of Alzheimer's disease [8] [9]–[11]. Recently, a meta-analysis also concluded that statins may provide a slight benefit in the prevention of Alzheimer's disease and all-type dementia [12]. However, a large-scale randomized controlled trial using atorvastatin treatment for 72 weeks failed to show any benefit on cognitive decline in those with mild to moderate Alzheimer's disease [13]. These unanticipated results underscored the need for further research on whether use of statins might benefit certain group of elderly subjects.

Given that many cross sectional study or database analysis reported a positive association between statin use and dementia, only a few of them was targeted at T2DM patients, and did not distinguish treatment outcomes between Alzheimer's disease and non-Alzheimer dementia. Therefore, we conducted this study by using the National Health Insurance Research Database (NHIRD) in Taiwan [14] to evaluate whether statin use can decrease the risk of dementia inT2DM cohort.

## Subjects and Methods

### Data sources

This study was designed as a population-based prospective study using the 1997–2008 Taiwan NHIRD. As of 2007, 98.4% of Taiwan's approximately 22.96 million population were enrolled in this program. The NHIRD database consists of four main files: the ambulatory expenditures by visit file (CD), the details of ambulatory care orders file (OO), the inpatient expenditures by admission file (DD) and the details of inpatient order file (DO). The source of data was drawn from the National Health Insurance Research Database (NHIRD), which was released for research purposes by the National Health Research Institutes (NHRI) in Taiwan, one of the largest and most comprehensive population-based databases in the world and is generally regarded as very accurate and complete.

The present study data were retrieved from one million randomly-sampled enrollee dataset from the mother NHIRD. This consisted of 1 million randomly selected subjects that represent about 4.5% of Taiwanese population from the entire NHI enrollee profile. There were no significant differences in age and sex between the 1 million random sampling dataset and the mother NHI research database [15]–[17]. Patient demographics in NHIRD included encrypted identification numbers, gender, date of birth and death, dates of admission and discharge, diagnostic data and procedures (up to five),and outcome at hospital discharge (recovered, died or transferred out). The diagnostic data included date of initial diagnosis, date of medical treatment, the International Classification of Diseases, Ninth Revision, Clinical Modification (ICD-9-CM) diagnosis code (up to five), and drugs code.

### Case selection and definition

In this study, we subdivided dementia into Alzheimer's disease and non-Alzheimer dementia. The cases of Alzheimer's disease included in this study were patients with at least 2 medical record diagnosis of Alzheimer's disease (ICD-9-CM codes 290.0, 290.10–290.13, 290.20, 290.21, 290.3, 294.1 and 331.0) from outpatients file in any 1 year during 2000–2008. The cases of non-Alzheimer dementia were comprised of patients who were diagnosed with dementia (ICD-9-CM codes 046.1, 290.1, 290.2, 290.4, 290.40–290.43, 294.11, 331.1, 331.11, 331.19, 331.2, and 331.7–331.9) at least twice in any 1 year between 2000 and 2008. A-code had been used by NHIRD until December 31, 1999; ICD-9-CM code has been employed since January 1, 2000. Since A-code cannot precisely identify subjects with Alzheimer's disease and non-Alzheimer dementia, therefore, we excluded these subjects with A-code (A210 and A222) before year 2000.

**Figure 1:**
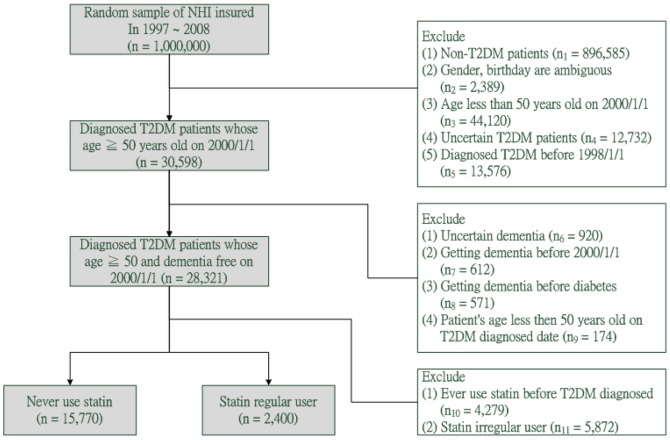
Study Subjects Selection Flow Chart.

Among 1 million representative sample of NHIRD from year 1997 to year 2008, we identified 28,321 T2DM patients who were above 50 years old with no history of dementia before January 1, 2000. T2DM was defined as 250.x0 and 250.x2 (ICD-9-CM), which presented in the CD file for at least 2 times within a year between 1998 and 2008 [18]; moreover, the diagnosis date of T2DM has to come before the diagnosis of dementia. The T2DM patients were then separated into two groups: those who had never used statins (n = 15,770) and those who used statins regularly (n = 2400). Both groups were studied with respect to their demographic characteristics and risk of developing dementia (Fig. 1). The definition of regular statin users was the regular use of statins for more than a year and the interval between successive prescription drug records cannot exceed 120 days. The cholesterol-lowering drugs classified as statins in this study include atorvastatin, fluvastatin, lovastatin, pravastatin, rosuvastatin, and simvastatin.

The comorbid medical conditions for each individual were evaluated by using the established Charlson-Deyo comorbidity index (CCI). CCI contains chronic diseases with a score between 1 and 6 points (1 point for myocardial infarction, congestive heart failure, peripheral vascular disease, cerebrovascular disease, dementia, chronic pulmonary disease, rheumatologic disease, peptic ulcer disease, mild liver disease, and diabetes without organ damage; 2 points for diabetes with organ damage, hemiplegia or paraplegia, severe renal disease, any malignancy including leukemia and lymphoma; 3 points for severe liver disease; 6 points for metastatic solid tumor and HIV infection), and the sum of these scores is regarded as the measurement of the burden of comorbidity. Chronic concomitant diseases of T2DM patients related to arthropathy, cardiovascular, gastrointestinal, hepatic, neoplastic, neurologic, pulmonary, and renal diseases were extracted out from the sum of CCI scores and has been described in details elsewhere [16], [19], [20].

The index date of follow up period for T2DM patients who never used statins were assigned to the date of T2DM diagnosis, whereas the index date of follow up period for T2DM patients who used statins regularly were assigned to the first prescription date of statins. The end date of follow up period for both non-statin users and regular statin users were assigned to the date of dementia, the date of death, or 31^st^ of December, 2008, whichever came first.

### Statistical analysis

The SAS statistical software was used to perform all programming and statistical analyses in this study. The demographic characteristics of T2DM patients who never used statins and T2DM patients with regular use of statins were compared by using χ2 for categorical variables and Wilcoxon rank-sum test as well as t test for continuous variables. Categorical variables such as age group, gender, types of stroke, CCI group, follow-up group, statin drug types, anti-diabetic drug types, and underlying chronic diseases were reported as percentages. Continuous variables such as the age, CCI score, and follow up period are reported as means and standard deviation.

Cox proportional hazards models were performed to estimate the hazard ratios in relation to T2DM patient with dementia and statin medication by adjusting for potential covariates, including age group, gender, CCI group, stroke types, and anti-diabetic drugs. The unjustified cumulative dementia-free probabilities from year 1997 to year 2008 were evaluated by Kaplan-Meier survival analysis. The log-rank test was used to compare the significance of inequality with respect to statin medication status curves.

The protocols had been submitted to our hospital's Institutional review board for review and approval was obtained.

## Results

### Demographic characteristics

Of the 28,321 patients diagnosed with T2DM whose age were above 50 and without history of dementia before 1^st^ January 2000, 15,770 were defined as patients who never used statins and 2,400 were defined as patients who regularly used statins. The mean (SD) age of the T2DM patients who used statins regularly were significantly younger than those who had never used statins (p<0.001). There were more women in regular statin users group and more men in non-statin users group. Statin users group had more stroke history than non-statin users group (p<0.001), mainly in ischemic stroke (p<0.001), transient ischemic stroke (p<0.001), and unclassified stroke (p<0.001). Moreover, T2DM patients with regular use of statins had higher comorbidities compared to those who never used statins. The mean (SD) follow-up time for T2DM patients with regular statin use were 3.9±1.9 years while non-statin users were 5.1±3.0 years (p<0.001). The distributions of regular statin medications, anti-diabetic drugs, score of CCI, profiles of associated chronic diseases were shown in Table 1.

**Table 1:** Demographics of study subjects according to their drug using status.

Descriptor	T2DM, but no statin^a^		T2DM, statin regular usage^b^		
Cases	15770	(%)	2400	(%)	p-value^c^
Age group \ Mean ± SD	67.0±8.8		65.8±7.4		<0.001
50 – 60	3901	24.7	616	25.7	<0.001
60 – 70	6231	39.5	1115	46.5	
70 – 80	4296	27.2	579	24.1	
≧ 80	1342	8.5	90	3.8	
Gender					
Male	8467	53.7	1042	43.4	<0.001
Female	7303	46.3	1358	56.6	
Stroke**^d^**	1528	9.7	393	16.4	<0.001
Hemorrhagic stroke	305	1.9	49	2.0	0.722
Ischemic stroke	898	5.7	267	11.1	<0.001
Transient ischemic stroke	306	1.9	83	3.5	<0.001
Unclassified	423	2.7	101	4.2	<0.001
Follow up group \ Mean ± SD	5.2±3.1		3.9±2.0		<0.001
0 – 2	3125	19.8	440	18.3	<0.001
2 – 4	3062	19.4	887	37.0	
4 – 6	2977	18.9	670	27.9	
6 – 8	2971	18.8	323	13.5	
8 – 10	2587	16.4	71	3.0	
≧ 10	1048	6.6	9	0.4	
Statin type**^e^**					
Atorvastatin			873	36.4	<0.001
Fluvastatin			247	10.3	<0.001
Lovastatin			394	16.4	<0.001
Pitavastatin			0	0.0	
Pravastatin			212	8.8	<0.001
Rosuvastatin			287	12.0	<0.001
Simvastatin			398	16.6	<0.001
Anti-diabetes drug type**^f^**					
Acarbose	7	0.0	138	5.8	<0.001
Metformin	151	1.0	725	30.2	<0.001
Thiazolidinedione (TZD)	1	0.0	46	1.9	<0.001
Sulfonylureas	194	1.2	947	39.5	<0.001
Meglitinide	12	0.1	101	4.2	<0.001
Insulin	1	0.0	32	1.3	<0.001
CCI group**^g^**\Mean ± SD	1.4±1.5		1.6±1.5		<0.001
CCI score 0	5329	33.8	627	26.1	<0.001
CCI score 1, 2	7475	47.4	1230	51.3	
CCI score 3, 4	2332	14.8	420	17.5	
CCI score ≧ 5	634	4.0	123	5.1	
Chronic diseases**^d^**					
Arthropathy	687	4.4	133	5.5	0.009
Cardiovascular	3791	24.0	690	28.8	<0.001
Gastrointestinal	2720	17.2	376	15.7	0.055
Hepatic	3583	22.7	727	30.3	<0.001
Neoplasm	496	3.1	79	3.3	0.703
Neurologic	108	0.7	24	1.0	0.090
Pulmonary	6539	41.5	998	41.6	0.913
Renal	1962	12.4	367	15.3	<0.001

a. Diagnosed type 2 diabetes mellitus (T2DM) patients who did not use statin before the end of follow up date.

b. The statin regular user is the patient who uses statin continuously at least over one year in the follow up time that nearby 2 statin prescriptions can't exceed 120 days.

This group of diagnosed T2DM patients is statin regular user group.

c. Wilcoxon rank sum test or Pearson's Chi-square test.

d. The case number is calculated before patient's index date.

The index date of patient who never uses statin before the end of follow up date is the T2DM diagnosed date.

The index date of patient who is statin regular user is the date of staring to take statin regularly.

e. The case number is the regular use of statin after patient's T2DM diagnosed date.

f. The case number is the regular use of the anti-diabetic drug before patient's index date.

g. Charlson comorbidity index (CCI): The diagnoses recorded in the National Health Insurance Research Database before the index date is used to calculate CCI score.

We exclude the diagnosis of diabetes mellitus and stroke from CCI score calculation, because these two disease entities are considered separately.

### Risk of dementia in T2DM patients by statin medication status

After adjusting for age group, gender, CCI group, stroke types and anti-diabetic drugs, results of the Cox proportional hazard model showed that the regular statin users had a lower risk for any dementia (adjusted HR: 0.6, 95% CI 0.42 – 0.88, p = 0.008) and Alzheimer's disease (adjusted HR: 0.48, 95% CI 0.30 – 0.76, p<0.001),than non-statin users but not for incident non-Alzheimer dementia (adjusted HR: 1.07, 95% CI 0.54–2.12 p = 0.844). We further classified regular statin users into three sub cohorts based on their average daily dose. The adjusted HR was 0.38 (95% CI 0.22–0.67, p<0.001) for the cohort prescribed with average daily dose of less than 10 mg. However, there were no significant differences with developing Alzheimer's disease in those prescribed with average daily dose between 10 mg and 20 mg, and for cohort prescribed with average daily dose greater than 20 mg as compared with non-statin users. Regular statin users showed no significant relationships with the risk of developing non-Alzheimer dementia both in unadjusted and adjusted results, nor in different doses prescribed. (Table 2). Analysis of different types of statin medications prescribed showed that T2DM patients who had received atorvastatin and simvastatin experienced significant lower risk of developing Alzheimer's disease than T2DM patients who never received any statin medications (Table S1). However, all types of statins had no protective effects on T2DM patients with non-Alzheimer dementia. (Table S1).

**Table 2:** Risk of Alzheimer's disease plus non-Alzheimer dementia, Alzheimer's disease and non-Alzheimer dementia among T2DM patients compared to diabetic subjects without statin.

Study subjects		Alzheimer's disease plus non-Alzheimer dementia					
Statin status	Mean daily dose (mg)^a^	Total	Dementia cases	Unadjusted HR (95% CI)	p-value	Adjusted HR (95% CI)^b^	p-value
No statin		15770	824	reference (1.0)		reference (1.0)	
Regular user		2400	53	0.58 (0.44 – 0.77)	<0.001	0.60 (0.42 – 0.88)	0.008
Cohort 1	<10	1407	28	0.50 (0.35 – 0.74)	<0.001	0.53 (0.34 – 0.83)	0.006
Cohort 2	10 – 20	667	16	0.65 (0.39 – 1.06)	0.083	0.68 (0.39 – 1.17)	0.164
Cohort 3	≧ 20	326	9	0.80 (0.41 – 1.54)	0.505	0.80 (0.39 – 1.64)	0.549

Study subjects		Alzheimer's disease					
Statin status	Mean daily dose (mg)^a^	Total	Dementia cases	Unadjusted HR (95% CI)	p-value	Adjusted HR (95% CI)^b^	p-value
No statin		15625	679	reference (1.0)		reference (1.0)	
Regular user		2388	41	0.54 (0.40 – 0.74)	<0.001	0.48 (0.30 – 0.76)	<0.001
Cohort 1	<10	1399	20	0.44 (0.28 – 0.68)	<0.001	0.38 (0.22 – 0.67)	<0.001
Cohort 2	10 – 20	664	13	0.64 (0.37 – 1.10)	0.106	0.57 (0.31 – 1.08)	0.084
Cohort 3	≧ 20	325	8	0.86 (0.43 – 1.73)	0.674	0.74 (0.34 – 1.61)	0.445
**Study subjects**		**Non-Alzheimer dementia**					
**Statin status**	**Mean daily dose (mg)**^a^****	**Total**	**Dementia cases**	**Unadjusted HR (95% CI)**	**p-value**	**Adjusted HR (95% CI)^b^**	**p-value**
No statin		15091	145	reference (1.0)		reference (1.0)	
Regular user		2359	12	0.73 (0.40 – 1.31)	0.290	1.07 (0.54 – 2.12)	0.844
Cohort 1	<10	1387	8	0.80 (0.39 – 1.63)	0.539	1.20 (0.54 – 2.66)	0.661
Cohort 2	10 – 20	654	3	0.67 (0.21 – 2.10)	0.491	0.96 (0.29 – 3.11)	0.939
Cohort 3	≧ 20	318	1	0.49 (0.07 – 3.51)	0.477	0.73 (0.10 – 5.45)	0.761

a. Mean daily dose (mg)  =  cumulative doses start from the regular taking drug date to the end of observation date/days between the start regular taking drug date and the end of observation date.

b. Adjust age group, gender, CCI group, stroke types and anti-diabetic drugs.

### Kaplan-Meier dementia-free survival curves

**Figure 2:**
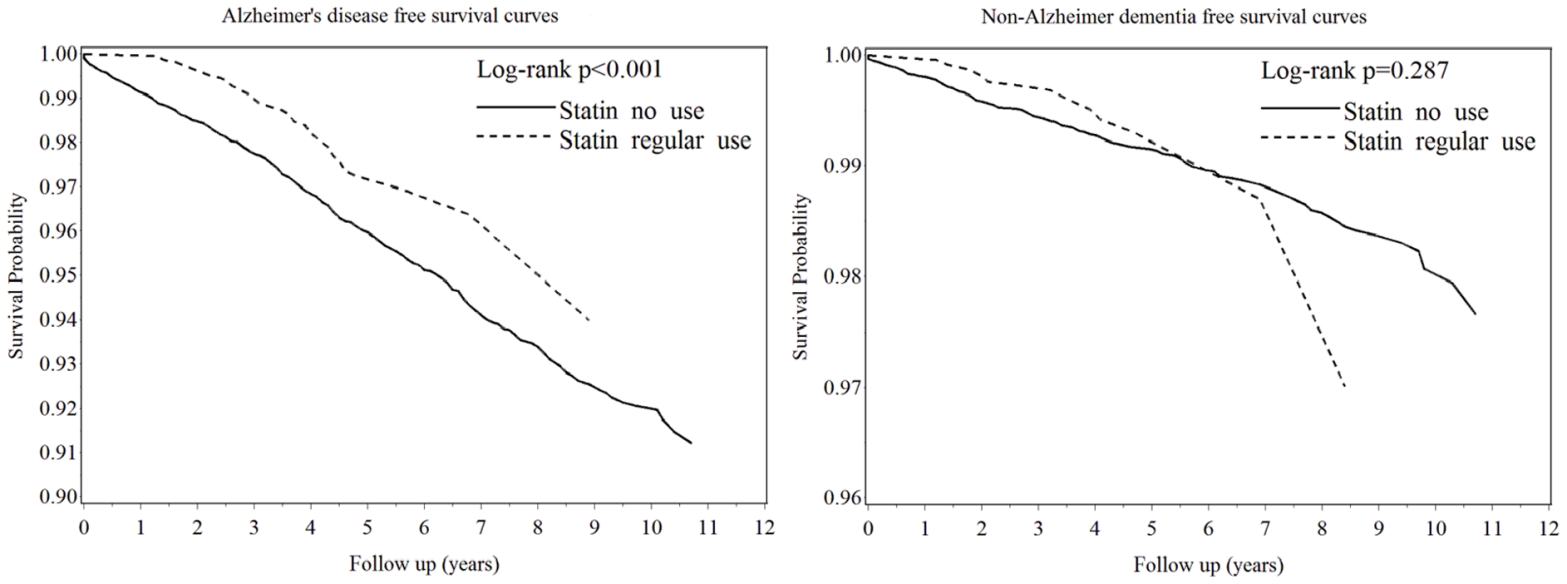
Survival Curves_ Alzheimer Disease and Non-Alzheimer Dementia.

The dementia-free survival rates for the T2DM cohort from year 1998 to year 2008 are presented in the Figure 2.Resutls of the log-rank test indicated that statin users had significantly higher Alzheimer's disease –free survival probability than non-statin users (p = 0.001). In contrast, no significant difference was found in non-Alzheimer dementia-free survival probability between statin users and non-statin users (p = 0.287).

## Discussion

The main finding of this study showed that, in a group of diabetes patients with age greater than 50, regular use of statins had lower risk of developing Alzheimer's disease dementia while it cannot decrease the risk of non-Alzheimer dementia. Our data confirmed the association between statin use and risk reduction of Alzheimer's dementia by several previous reports [7], [21]–[23]. But their cohort did not specifically target T2DM patients and their patient numbers were also relatively smaller compared to our study. There were 2 previous studies that examined statin use and dementia in T2DM cohort. Fei et al. [24] reported that statin use was associated with decreased risk of all cause dementia including Alzheimer's dementia and vascular dementia in a group of Chinese elderly diabetes subjects. By using a national cohort of beneficiaries of the Department of Veterans Affairs, Johnson et al [25] also reported that statin use can reduce risk of dementia in their diabetic cohort in additional to certain antihypertensive medications. However, this latter study did not distinguish Alzheimer's dementia from non-Alzheimer dementia. There are some studies with opposite result. Li G et al. [23] reported that, by examining brain tissue, antecedent statin use was not associated with neurofibrillary tangle change at autopsy [26], [27]. In the Cardiovascular Health Study, ever use of statins was not associated with the risk of all-cause dementia, Alzheimer disease alone, mixed Alzheimer disease and vascular dementia, or vascular dementia alone while current use of statins was associated with significant lower risk of all-cause dementia and any Alzheimer disease [27]. Very recently, a large-scale randomized controlled trial using atorvastatin 80 mg/d therapy for 72 weeks failed to show benefit in cognitive function decline in a group of elderly with mild to moderate Alzheimer disease [13]. These findings suggest that the benefit of statin might either be limited to certain subgroup (such as diabetes, as found in our data) or might require further analysis.

In our present study, we did not show a risk reduction of non-Alzheimer dementia in response to regular statin use. In fact, vascular dementia accounts for about 20–30% of all the dementia and comprises the majority of non- Alzheimer's disease dementia [28]. There have been inconsistent reports about whether circulating lipid levels contribute to development of dementia. In support of our findings, Reitz et al. [29] reported that there was only a weak relation between non-HDL-C, LDL-C, and HDL-C levels and the risk of vascular dementia.

In our study, the most lipophilic simvastatin [30] and the second lipophilic atorvastatin were effective in reducing the risk of Alzheimer's disease dementia after adjustment for several confounding factors. Other less lipophilic statins (fluvastatin, pravastatin, lovastatin, rosuvastatin) showed only a trend towards a decreased risk but did not reach statistical significance. Using US Veterans Affairs database, Wolozin et al [31] reported that atorvastatin was associated with only a trend towards modest reduction in the incidence of dementia. Sparks et al [32] showed that treatment with atorvastatin had a beneficial effect on cognition and behavior in patients with mild to moderate Alzheimer's dementia. All their findings have not been confirmed by the recently published randomized controlled, LEADe study [13]. Wolozin et al. [31] reported that the use of simvastatin was associated with a strong reduction in the incidence of dementia. In MRC/BHF Heart Protection Study, simvastatin has not been confirmed to have a clinically demonstrable cognitive benefit in the treatment of Alzheimer's dementia [33]. In the PROSPER study, Shepherd et al. [34] reported that pravastatin had no significant effect on cognitive function or disability after following up for 3.2 years. Although our findings might favor the protective effect of statin with the characters of lipophilicity, investigators in the Rotterdam Study indicated that the protective effect of statins on Alzheimer's disease was independent of the lipophilicity of statins [22].

The greatest strength of our study lies in its large scale population and powerful statistical analysis. lt was based on pharmacy and medical claims that included patients from throughout Taiwan so the results can be generalized for an insured population. Another strength is the relatively longer follow up period compared to previous studies. There are some important limitations in our study. Our administrative claims data set did not provide relevant clinical details about status of glycemic and blood pressure control, co-medication use, other potential risk factors for dementia such as apolipoprotein E4 genotype, education, diet, smoking, and alcohol use. In addition, our claims data followed up for 8 years, which might be insufficiently long for development of dementia. In addition, patients may have received medications from sources that are not captured by pharmacy claims. Finally, our patients are all Taiwanese people; it is unclear whether our results would apply to other ethnic groups.

## Conclusions

Our study found that regular use of statins can decrease the risk of developing Alzheimer's disease but not non-Alzheimer dementia in patients with T2DM. Among those statins, atorvastatin and simvastatin at dose of 10 mg per day showed the most significant protection. Our findings raise interesting possibilities about the role of statins in delaying the onset of dementia in patients with T2DM. Further investigations are necessary to clarify such association between statin use and risk of dementia in patients with T2DM.

## Supporting Information

Table S1 **HR_By Drug Type.**
(DOCX)
