# Correction: Effects of Statins on Incident Dementia in Patients with Type 2 DM: A Population-Based Retrospective Cohort Study in Taiwan

**DOI:** 10.1371/journal.pone.0100019

**Published:** 2014-06-09

**Authors:** The PLOS ONE Staff

There is an error in the affiliations for co-author, Jorng-Tzong Horng. The correct affiliations for Jorng-Tzong Horng are:

^3^ Department of Biomedical Informatics, Asia University, Taichung, Taiwan

^6^ Department of Computer Science and Information Engineering, National Central University, Chungli, Taiwan
