# Supplementary figures and images for: Effects of Statins on Incident Dementia in Patients with Type 2 DM: A Population-Based Retrospective Cohort Study in Taiwan
Source: PLoS One. 2014 Feb 10;9(2):e88434. doi: 10.1371/journal.pone.0088434 (PMC3919769; doi:10.1371/journal.pone.0088434)

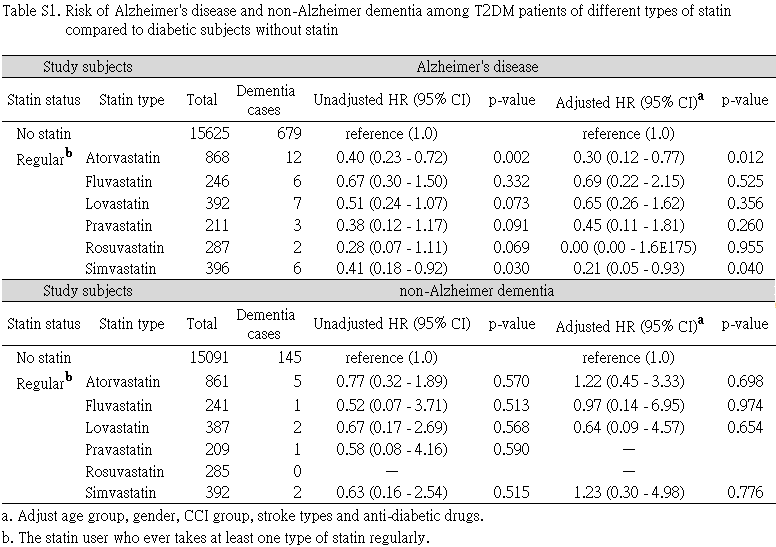

Supplement: Table S1 — HR_By Drug Type. (DOCX) [file pone.0088434.s001.docx]
